# Benefit of dosimetry distribution for patients with multiple brain metastases from non-small cell lung cancer by a Cyberknife stereotactic radiosurgery (SRS) system

**DOI:** 10.1186/s12885-020-07624-4

**Published:** 2020-11-25

**Authors:** Xuyao Yu, Yuwen Wang, Zhiyong Yuan, Hui Yu, Yongchun Song, Lujun Zhao, Ping Wang

**Affiliations:** 1 Department of Radiation Oncology and Therapy, Tianjin Medical University Cancer Institute and Hospital, 60 Weijin Road, Hexi, Tianjin, China; 2 Department of Radiotherapy, Konggang Branch of Tianjin Cancer Hospital, Dong Fifth Road, Dongli District, Tianjin, China; 3 Biomedical Engineering, Tianjin University, Weijin Road, Nankai District, Tianjin, China

**Keywords:** Cyberknife, Multiple brain metastases, Single-lesion plans, Multiple-lesions plan

## Abstract

**Background:**

In order to obtain a high dose conformal index of tumor and steep dose fall-off in healthy tissues for brain metastasis stereotactic radiosurgery (SRS), the aim of this study was to investigate SRS planning optimization by comparing one multiple-lesions plan (MLP) with multiple single-lesion plans (SLPs) for patients with multiple brain metastases using the Cyberknife (CK) system.

**Methods:**

Fifty non-small cell lung cancer (NSCLC) patients (28 males and 22 females) with 2–4 brain metastases, inter-tumour distances less than 3 cm, were retrospectively replanned with the original prescription dose (12–32 Gy) in the original fractions (1–3). Two different clinical CK SRS plans (SLPs and MLP) were generated for the same patients with the same collimator and prescription isodose line (62–68%) by the CK Multiplan System. Both SLPs and MLP were able to achieve > 95% PTV volume covered prescription dose and met the Timmerman 2011 organs at risk (brainstem, optic nerve and pituitary) constraints.

**Results:**

Compared with those in the SLPs, the maximum dose (*D*_*max*_) and mean dose (*D*_*mean*_) of brainstem in the MLP were reduced 0.22–3.13% (2.62%) and 2.71–12.56% (5.57%), respectively, all *P <* 0.05. Meanwhile, the volumes of the whole brain minus the tumors that received a single dose equivalent of 8–16 Gy (V8Gy-V16Gy) were effectively reduced in the MLP. The treatment time parameters, the total number of beams and monitor units, of the MLP were reduced by 3.31 and 1.47% (*P <* 0.05), respectively. Although there were a few differences in the conformity index (*CI*) and homogeneity index (*HI*) between the two treatment plans, the differences were not statistically significant (*P* = 2.94 and 1.08 > 0.05).

**Conclusion:**

One multiple-lesions plan for brain metastases could achieve higher precision in the target and lower doses in healthy tissue while shortening the treatment time and improving the treatment efficiency over multiple single-lesion plans.

## Background

Brain metastases develop in 20–40% of cancer patients, including 36–64% of patients with lung cancer, 15–25% of patients with breast cancer, and a few patients with melanoma, colorectal cancer and renal carcinoma [1, 2]. Clinical treatment options for patients with brain metastases include surgery, whole brain radiotherapy, and stereotactic radiosurgery (SRS) alone or in combination [3]. Although whole brain radiotherapy (WBRT) can improve the control rate of multiple brain metastases, it cannot prolong the overall survival of patients, and it increases the toxic side-effect risk in the patients’ nervous system [4–6]. However, SRS alone or combined with immunotherapy can guarantee a curative effect and avoid long-term adverse reactions to clinical treatment. Therefore, this combination has been recognized and accepted by an increasing number of clinical experts and patients, and it has gradually become the core protocol to treat multiple metastases.

The American National Comprehensive Cancer Network (NCCN) recommends SRS for patients with brain metastases that meet the following criteria: 1) tumour diameter < 5 cm, 2) no more than 4 tumours, 3) co-application of surgery or WBRT, and 4) recurrence after SRS (6 months) [7]. Common SRS treatment equipment includes Gammaknife, Cyberknife (CK) and various kinds of linear accelerators [8, 9]. CK can be used to treat encephalic lesions by adapting orthogonal X-ray tubes to image the patient’s head in real time and performing radiotherapy of the lesions by positioning the CK after registering the patient’s skull images. Therefore, CK SRS can improve the dose delivered to the tumour and simultaneously reduce injury to the healthy brain tissue [10].

In addition to local control, remote control and overall survival rate, various nervous functional states and quality of life indicators should be the focus in brain metastasis treatment. CK SRS performs single large-segmentation radiotherapy for treating patients with multiple brain metastases. This easily results in a greater radiation dose absorbed by healthy brain tissue and organs at risk (OAR) adjacent to the tumours and increases the probability of clinical cerebral oedema, delayed intratumoral bleeding or brain necrosis [11, 12]. Therefore, effectively reducing the irradiation dose absorbed by normal brain tissue is essential for CK SRS treatment planning.

Single-lesion plans (SLPs), which are one-on-one treatments for SRS plans and tumours, are often used during CK SRS planning for patients with multiple metastases. Brain tissue around the gross tumour volume (GTV) receives high radiation dose in a short time, because CK SRS uses higher single dose and less fraction. Therefore, when the tumours are very close together (outer contour distance < 3 cm), the radiation dose delivered to the healthy brain tissue between tumours may be larger than the prescription dose and must be refocused in multiple plan composition. In this paper, we aimed to develop a multiple-lesions plan (MLP) that used only one plan for the treatment of multiple brain metastases. Retrospective analysis and evaluation were performed on the quality, efficiency and differences of dose distribution between CK SRS SLPs and MLP for 50 non-small cell lung cancer (NSCLC) patients with multiple metastases in the CK centre of Tianjin Medical University Cancer Hospital to provide a reference basis for the clinical design of CK SRS treatment plans.

## Methods

### Patients and simulation

Between January 2016 and June 2019, 50 patients with 2–4 brain metastases form non-small cell lung cancer, inter-tumour distances less than 3 cm, were treated at Tianjin Medical University Cancer Institute and Hospital using the same prescription dose in the same fractions by CK system (Cyberknife III, Accuray Inc., Sunnyvale, CA). All patients had no other distant metastasis and did not recieve pre-CK SRS metastasectomy or pre- or concurrent-to-CK SRS WBRT. Karnofsky Performance Scores (KPS) score of 26 patients was less than 70, and all patients were in RTOG-RPA class 2 or 3. This report compared the different dosimetry profiles in different plans for the same patient by the Multiplan system (Accuray, Sunnyvale, CA, USA). Table 1 shows the patient characteristics.

**Table 1:** 50 Patient characteristics

	Number *(%)*
Age (years)	
Age ≥ 60	27 (54.0%)
Age<60	23 (46.0%)
Sex	
Male	28 (56.0%)
Female	22 (44.0%)
KPS score	
KPS ≥ 70	24 (48.0%)
KPS < 70	26 (52.0%)
Location of tumor	
Cerebral hemisphere	66 (57.9%)
Cerebellum	48 (42.1%)
Tumor number	
2	39 (78.0%)
3	8 (16.0%)
4	3 (6.0%)
Tumor volume	
<1 cc	23 (20.2%)
1–5 cc	84 (73.7%)
5–10 cc	7 (6.1%)
Tumor diameter	
<1 cm	26 (22.8%)
1–3 cm	77 (67.5%)
>3 cm	11 (9.6%)

All patients were positioned supine on the couch and fixed by a custom-fitted thermoplastic mask. The computed tomography (CT; Philips Brilliance Big Bore CT, Netherlands) and enhanced T1-weighted magnetic resonance imaging (MRI; Siemens Magnetom 1.5-T, Siemens AG Medical Solutions, Germany) was performed to scan throughout brain. CT scanning was performed with 120 Kv, 320 mAs, pitch 1.15 and reconstruction slice 1.5 mm. The MRI images obtained from T1-weighted magnetic resonance imaging scans obtained, with 5126 × 512 matrix and 1.5 mm reconstruction slice. CT and MRI images were fused and used to delineate the gross tumour volume (GTV) and organs at risk (OARs), including the brainstem, eyes, lenses, optic nerves, optic chiasm, and pituitary gland. The plan tumour volume (PTV) was created by added a 1.6-mm margin to the GTV, following the targeting error of the brain CK SRS under skull tracking is 0.956 mm, except for patients with brainstem metastasis.

### CK SRS treatment planning

Brain metastases that were close to each other and required the same prescription radiation dose and fractions could be planned together. Five dose-limiting shells (2 mm, 3 mm, 5 mm, 7 mm, 9 mm) were placed away from each PTV to limit the dose distribution in healthy brain tissues. The CK SRS plans were designed and optimized by the CK Multiplan system, based on the delineation results and requirements. Two different treatment plans were designed for every patient, including multiple SLPs (one plan to one PTV) and MLP (one plan for all PTVs). When SLPs were used to treat the patients, the different treatment plans for the different lesions were executed by sequential single-plan therapy. The same collimator and prescription isodose (65–70%) were adopted during plan designing for the same patient without the iris or MLC system to ensure consistency of the beam data in the plans. All the plans be designed meeting the requirement, included > 95% coverage of the PTV with the prescription dose and the Timmerman 2011 OAR (brainstem, optic nerves and pituitary) constraints [13]. The radiotherapy path was not allowed to pass through the patients’ lens in all the plans, so the maximum dose of lens was < 1 Gy. For the plans evaluation step, high-resolution calculation was performed to finalize CK SRS plans.

### Data analysis

The same patient’s SLPs, with the same prescription dose and the same fractions, were fused and evaluated by the plan QA functional component (Reference Plan) in the Cyberknife Multiplan system. The dosimetry distribution in the metastatic tumours, normal brain tissue and OAR were compared between MLP and SLPs for each patient. The conformity index (*CI*) represents an objective measure of how well the distribution of radiation follows the shape of the PTV and is calculated as follows:

1 $$ CI=\mathrm{PIV}/\mathrm{TIV} $$

where PIV and TIV are the volume included by the prescription isodose and the tumour volume covered by the prescription isodose, respectively. This *CI* is different with the radiation therapy oncology group (RTOG) definition, which is PIV divided by total tumour volume [14]. The *CI* value close to 1 indicated a good plan.

To quantify the difference in the dose parameter, the values of the minimum dose (*D*_*min*_), maximum dose (*D*_*max*_) and covering mean dose (*D*_*mean*_) for the healthy brain tissue and OAR were expressed as a percentage of the global maximum dose in the plans. Their reduction is calculated as follows:

2 $$ R=\left({Data}_{SLP}-{Data}_{MLP}\right)/{Data}_{MLP} $$

where *Data*_*SLP*_ and *Data*_*MLP*_ represent the values in the SLPs and MLP, respectively.

Furthermore, the volumes of the whole brain deducted the PTVs received a single dose equivalent of 4 to 16 Gy (V4Gy-V16Gy) and were evaluated by assuming α/β ratio of 2.0 to the brain tissue with iLQ (V4.0) [15]. The total number of beam and monitor units (*MUs*) in plans were compared.

All statistical analyses were performed with SPSS (Statistical Product and Service Solutions for Statistical Computing, IBM, USA, version 19.0). Data from different plans were compared with a two-sided paired *t* test. A *P*-value < 0.05 was considered statistically significant.

## Results

**Fig. 1:**
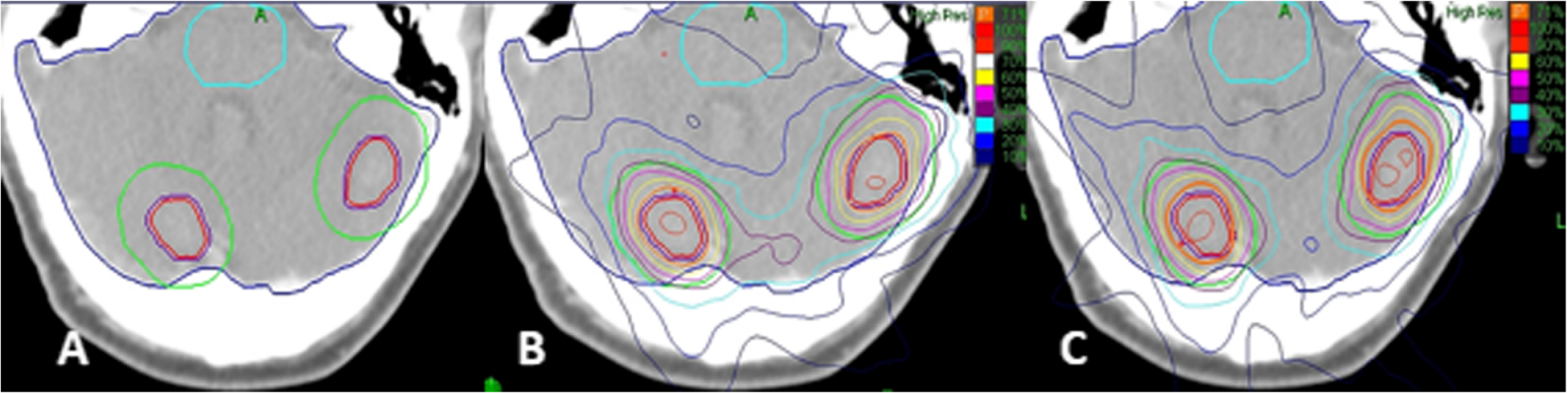
Different CK SRS plans for the same patient with multiple brain metastases. The representative patient had axial images taken, (**a**) shown the location of the tumors (red line area), healthy brain tissue around the PTVs (green line area), the whole brain (blue line area) and OAR (Light blue line area was brainstem), (**b)** and (**c**) were single-lesion-plan (SLP) and multi-lesions-plan (MLP). The red and purple lines indicate the GTV and the PTV, respectively. Black and blue lines represent brain tissue and brainstem. Green contour was covered with 6-mm thick zone adjacent to the PTV. Isodose: Orange-Prescription dose, Red-100 and 90%, White-70%, Yellow-60%, Pink-50%, Purple-40%, Blue represents 10 to 30 from light to deep, respectively

Figure 1 shown the dosimetry distribution of the MLP versus the SLPs for the same patient with two brain metastases. The results indicate that the radiation around the PTV was more widespread or the SLPs; for example, the 40% isodose (purple line) was included in the PTV + 6 contour in the MLP (as shown in Fig. 1(b)) but not in the SLPs (as shown in Fig. 1(c)). While the OAR (brainstem) were very well protected and were characterized as less irradiated areas, the 10% isodose did not appear in the brainstem from the MLP. These results indicate that using an MLP for patients with multiple brain metastases could significantly reduce the dose distribution in healthy brain tissue and OAR.

To quantify the difference in dosimetry distribution between the two plans, statistical analysis was carried out on the CK SRS plans for the 50 patients, as shown in Table 2. The values of *D*_*min*_, *D*_*max*_ and *D*_*mean*_ are expressed as percentages of the global maximum dose in the plans. Compared with those in the SLPs, in the MLP, the dose in PTV + 6 significantly decreased, the *D*_*max*_ value dropped 0.29–1.91%, the *D*_*mean*_ value decreased 1.89–2.58%, and the *D*_*min*_ value dropped 4.35–8.98%. The dose parameters of the OAR are shown in Table 2. MLP decreased the radiation dose in the OAR with respect to the dose from the SLPs; specifically, the *D*_*max,*_ and *D*_*mean*_ value of the brainstem decreased by 0.22–3.13% and 2.71–12.56%, respectively. Thus, using an MLP for treating patients with multiple brain metastases could reduce the risk of symptomatic radiation-induced injury in healthy brain tissue and OAR.

**Table 2:** Dosimetric distribution of PTV + 6 and OAR in CK SRS SLP and MLP for 50 patient

	SLP mean (range)	MLP mean (range)	*R* mean (range)	*t*	*p*
PTV + 6					
*D*_*Max*_	89.75%(80.20–93.90%)	89.07%(79.99–92.11%)	0.77%(0.29–1.91%)	− 1.00	0.04
*D*_*mean*_	54.69%(46.54–65.02%)	53.18%(45.34–63.79%)	2.34%(1.89–2.58%)	2.10	0.03
*D*_*Min*_	35.46%(29.65–43.55%)	33.82%(28.36–39.64%)	4.62%(4.35–8.98%)	5.40	<0.01
Brainstem					
*D*_*Max*_	21.57%(18.15–23.76%)	21.02%(18.49–23.04%)	2.62%(0.22–3.13%)	2.15	0.03
*D*_*mean*_	11.49%(5.27–20.68%)	10.85%(4.45–20.12%)	5.57%(2.71–12.56%)	1.18	<0.01
Optic Nerve					
*D*_*Max*_	14.10%(4.56–18.85%)	13.56%(4.44–18.57%)	2.63%(1.49–3.83%)	3.66	0.02
*D*_*mean*_	7.16%(5.18–10.02%)	6.88%(4.52–9.35%)	6.91% (3.69–12.74%)	−5.28	<0.01
Optic Chiasm					
*D*_*Max*_	14.90%(7.53–21.05%)	14.41%(7.47–19.93%)	3.29% (0.80–5.32%)	−3.77	0.04
*D*_*mean*_	6.08%(3.21–8.96%)	5.92%(2.95–8.83%)	5.63% (1.45–8.10%)	6.07	<0.01

PTV + 6 was 6-mm-thick zones of healthy brain tissue adjacent the PTVs, *D*_*Max*_, *D*_*mean*_ and *D*_*Min*_ were maximum dose, mean dose and minimum dose, expressed as percent of the global maximum dose in plans

**Fig. 2:**
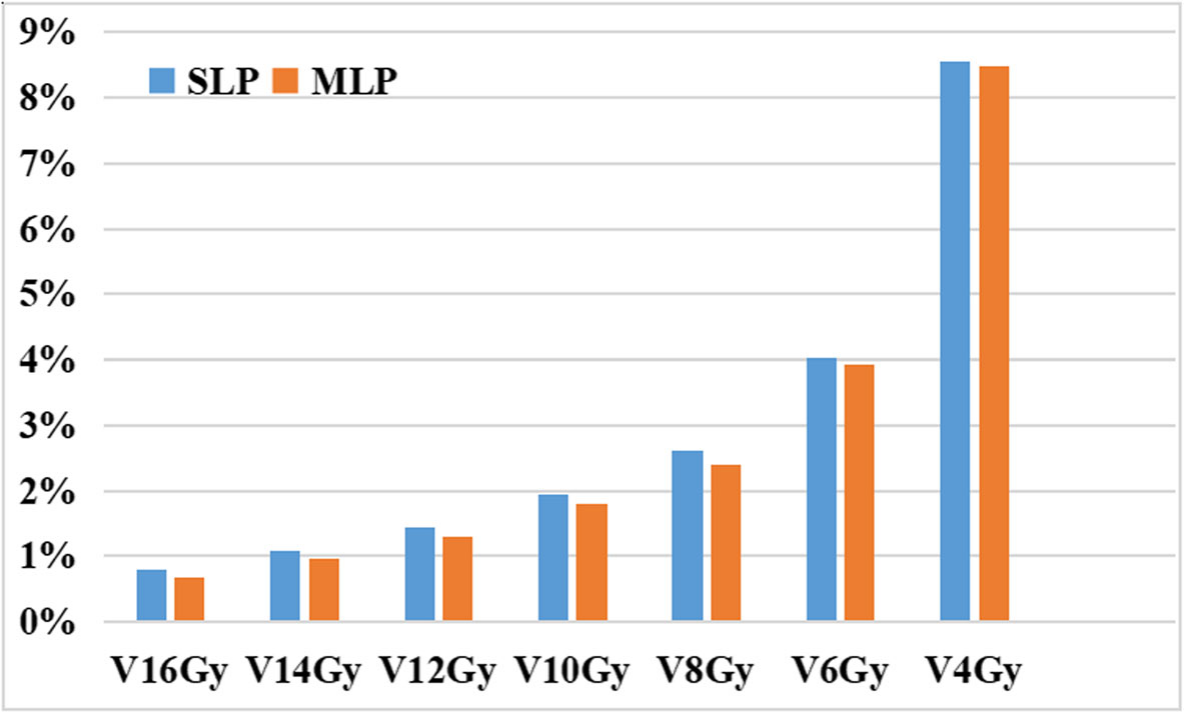
The statistical results of V4Gy-V16Gy in the whole brain tissue excluding the PTVs

The statistical results for V4Gy-V16Gy were shown in Fig. 2. Although there was no obvious difference for V4Gy and V6Gy, compared with those in the SLPs, the V8Gy-V16Gy values showed a marked decrease in the MLP. This finding provides more evidence for the theory that using an MLP could protect healthy brain tissue better than multiple SLPs while satisfying the need for clinical treatment.

Table 3 compares the CK SRS plan parameters. The conformity index (*CI*) and homogeneity index (*HI*) values of the two different plans were similar, showing no significant difference. This result indicated that either the SLPs or the MLP could yield desirable curative effects for the PTV getting prescription dose homogeneously of the patients clinically. However, the total number of beam and monitor units (*MUs*) were lower in the MLP, with average decreases of 4.63 and 0.56%, respectively. Radiotherapy linear accelerator is carried to different beam paths by the manipulator and outputs prescription dose (through *MUs*). Therefore, the execution time (including moving time and output time) of the MLP was apparently less than that of the SLPs during multiple brain metastases patients treatment by CK SRS.

**Table 3:** Indexes of 50 patient CK SRS plans

	SLP mean (range)	MLP mean (range)	*R* mean (range)	*t*	*P*
Conformity index (*CI*)	1.14 (1.04–1.23)	1.13 (1.03–1.24)	/	1.57	2.94
Homogeneity index (*HI*)	1.35 (1.32–1.47)	1.37 (1.33–1.47)	/	−4.21	1.08
Total beam number	133 (101–149)	128 (97–138)	3.96% (3.76–6.71%)	−1.71	<0.01
Total monitor units (*MU*)	12,774.83 (9121.67–15,243.74)	12,719.26 (8974.16–15,158.49)	0.56% (0.43–1.64%)	1.09	0.04

## Discussion

With the improvement of medical technology and increasingly prolonged lifespans of cancer patients, brain metastasis has become an important clinical manifestation for late cancer patients. Some researchers have shown that approximately 80% of patients with brain metastases could have 1–3 metastatic tumours simultaneously [16]. At present, treatment methods for brain metastatic tumours mainly include surgery, WBRT, SRS, etc. [17]. Chang et al. believe that compared with WBRT, SRS could increase the local control rate of intracranial tumours, avoid the occurrence of long-term nerve cognitive disorder and improve patients’ quality of life [18]. Rades Dirk et al. performed a retrospective comparison and analysis on patients with brain metastases treated by SRS or WBRT (number of tumours< 3 and tumour diameter < 4 cm), and the results showed that the median survival and one-year local control rate in the SRS group were superior to those in the WBRT group [19, 20]. Chung C et al. indicated that 68% of radiation oncologists believed 1–3 brain metastases were ideal quantities to be treated with stereotactic radioactive surgery (SRS) [21]. Murovic Judith et al. performed a comparison and analysis on patients with 1–3 brain metastases and on the curative effect for patients with more than 4 brain metastases who only received CK SRS. The results showed that the mean overall survival (OS) for both was 13 months, with no significant difference; therefore, CK radiosurgery could effectively control patients with brain metastases [22].

For the treatment of multiple brain metastases, CK SRS can effectively shorten the treatment cycle and duration. As a result, severe radiation damage in the clinic can be avoided. Additionally, the local control rate of patients’ lesions can be improved. In this study, analytical research was performed on 50 patients with multiple brain metastases treated by SLPs and an MLP from the CK SRS system. The results showed that the MLP could ensure that the prescription dose covered the PTV, effectively reducing the dose distribution to healthy brain tissue and OAR. The *CI* and *HI* value of the SLPs and MLP for the same patient were similar, showing no significant differences. Thus, these two different plans could lead to a relatively ideal dose distribution for the PTV. Radiotherapy with an MLP for patients with multiple brain metastases can reduce the radiation doses delivered to the healthy brain tissue between tumors, especially functional areas. This was an effective way to improve the well-being and overall health of patients after radiotherapy. Furthermore, because the robot arm controlling in CK system must move to each beam during radiotherapy, when the exposure interval for the target location was set, the number of total beam number and total monitor units would determine the treatment time in the Cyberknife G3 system. Compared with the SLPs, the MLP with fewer treatment beam and total monitor units could effectively shorten the treatment time. Therefore, the MLP, shortened treatment time and lower treatment costs, could be used for good clinical treatment of patients with multiple brain metastases by CK SRS.

It is worth noting that a multiple collimator (≤ 3 collimators) combination is needed to realize the MLP for CK SRS treatment when the patient has different sizes and volumes of metastatic lesions. However, a technician is needed to change the collimators when a third generation or lower version of the CK system (G3) is used to implement clinical treatment, which can cause certain complications and extend the treatment time. Therefore, both the convenience and safety of clinical operation should be comprehensively considered during CK SRS planning.

In summary, when CK SRS is used for the clinical treatment of patients with multiple brain metastases, adopting an MLP can ensure dose distributions and curative effects, effectively reducing radiation injury to the patient’s normal brain tissue and OAR and shortening the duration of clinical treatment. CK SRS plans are designed for patients with multiple brain metastases, and dose distributions and clinical treatment time should be fully considered. However, the clinical curative effects for both plan designs must be further discussed by collecting and tracing more cases to establish a reliable clinical database, providing a more significant reference for the implementation of treatment.

## Conclusion

The results of this study demonstrated that the multiple-lesions-plan (MLP), with a lower total number of beam and monitor units, significantly reduced radiation injury outside the tumours relative to the SLPs. The resultant maximum dose (*D*_*max*_) and mean dose (*D*_*mean*_) value of the OAR (brainstem) in the MLP were significantly lowered. Only one multiple-lesions plan is needed to meet the needs of SRS treatment for multiple brain metastases, deliver a large dose to the tumour and minimize the amount of radiation delivered to healthy tissues.

## Data Availability

The data-sets used and/or analyzed during the current study are available from the corresponding author on reasonable request.

## Abbreviations

SRS: Stereotactic radiosurgery
WBRT: Whole brain radiotherapy
NCCN: National comprehensive cancer network
CK: Cyberknife
OAR: Organ at risk
SLP: Single-lesion-plan
MLP: Multiple-lesions-plan
NSCLC: Non-small cell lung cancer
GTV: Gross tumor volume
PTV: Plan tumor volume
*CI*: Conformity index
*HI*: Homogeneity index
RTOG: Radiation therapy oncology group
*D*_*min*_: Minimum dose
*D*_*max*_: Maximum dose
*D*_*mean*_: Covering mean dose for healthy brain tissue
V4Gy-V16Gy: The volumes of the whole brain minus the PTVs received a single dose equivalent of 4 to 16 Gy
*MUs*: Monitor units
